# The Seasonal Fluctuation of Fatigue in Multiple Sclerosis

**DOI:** 10.3389/fneur.2022.900792

**Published:** 2022-06-17

**Authors:** Matthias Grothe, Stefan Gross, Marie Süße, Sebastian Strauss, Iris Katharina Penner

**Affiliations:** ^1^Department of Neurology, University Medicine Greifswald, Greifswald, Germany; ^2^Department of Internal Medicine B, University Medicine Greifswald, Greifswald, Germany; ^3^DZHK (German Center for Cardiovascular Research), Partner Site Greifswald, Greifswald, Germany; ^4^Department of Neurology, Medical Faculty Heinrich Heine University Düsseldorf, Düsseldorf, Germany; ^5^COGITO Center for Applied Neurocognition and Neuropsychological Research Düsseldorf, Düsseldorf, Germany; ^6^Department of Neurology, Inselspital, Bern University Hospital, University of Bern, Bern, Switzerland

**Keywords:** multiple sclerosis, seasonal, fatigue, sun, neuropsychological

## Abstract

**Background:**

Fatigue is a common symptom in patients with multiple sclerosis. Several studies suggest that outdoor temperature can impact fatigue severity, but a systematic study of seasonal variations is lacking.

**Methods:**

Fatigue was assessed with the Fatigue Scale for Motor and Cognitive Functions (FSMC) in a temperate climatic zone with an average outdoor temperature of 8.8°C. This study included 258 patients with multiple sclerosis from 572 visits temporally distributed over the year. The data were adjusted for age, sex, cognition, depression, disease severity, and follow-up time. Linear regression models were performed to determine whether the temporal course of fatigue was time-independent, linearly time dependent, or non-linearly time dependent.

**Results:**

Fatigue was lowest during January (mean FSMC: 49.84) and highest during August (mean FSMC: 53.88). The regression analysis showed the best fit with a model that included months + months^2^, which was a non-linear time dependency. Mean FSMC per month correlated significantly with the average monthly temperature *(*ρ = 0.972; *p* < 0.001).

**Conclusion:**

In multiple sclerosis, fatigue showed a natural temporal fluctuation. Fatigue was higher during summer compared to winter, with a significant relationship of fatigue with outdoor temperature. This finding should be carefully taken into account when clinically monitoring patients over time to not interpret higher or lower scores independent of seasonal aspects.

## Introduction

Multiple sclerosis (MS) is a chronic inflammatory disease of the central nervous system, characterized by inflammation, degeneration, axonal damage, and demyelination (1, 2). Among the heterogenous symptoms of MS, fatigue is common, with a reported prevalence of about 90% (3–6). Increased fatigue in MS is associated with impaired quality of life (7), reduced vocational status (8), and suicidal ideations (9). The underlying pathophysiology of fatigue in MS remains poorly understood with various studies suggesting immunological, neuroanatomical, and psychological causes (10, 11). In clinical practice, the evaluation of fatigue is difficult, due to its interactions with overall disability and other neuropsychological impairments (10, 12). Especially the highly prevalent mood disturbances as well as cognitive impairments in MS may confound objective assessment (6, 12). Besides, many patients report a general worsening due to heat exposure, known as Uhthoff's phenomenon (13). In addition to these patient-specific variables, environmental factors, like outdoor temperature, may also influence fatigue severity. Like another common neuropsychological symptom, depression, it seems at least plausible that fatigue might be inversely associated with sun exposure (14, 15). However, a majority of patients have reported that fatigue worsens with heat (4, 16). Nevertheless, a previous serial assessment of 45 patients with MS reported that outdoor temperature had no effect on fatigue (17).

Here, we analyzed real-world data of a cohort of patients with MS to test whether fatigue in MS was time-dependent. We adjusted our analysis for potential interacting variables, including age, sex, cognition, depression, disease severity, and follow-up time. Based on the literature and the presumptions, time dependency can be parameterized in three different ways – a linear course, an increasing during the summer compared to the winter, or the inverse course with increasing during winter and decreasing during summer. Therefore, we constructed several types of regression models and determined which model fit best to our fatigue data.

## Materials and Methods

### Participants

This retrospective cohort study was approved by the local ethics committee of the University of Medicine in Greifswald (BB221/20). Medical reports from the MS outpatient clinic between January 2017 and September 2021 were analyzed. Patients were enrolled when data on all variables of interest were available: date, age, sex, medication, disability score from the Expanded Disability Status Scale (EDSS) (18), depression score from the Beck Depression Inventory (BDI) (19), fatigue score from the Fatigue Scale for Motor and Cognition (FSMC), and information processing speed from the Symbol Digit Modalities Test (SDMT) (20). All data were collected during the clinical visits. All patients are living in Mecklenburg-Vorpommern, in the north-east of Germany next to the Baltic sea. Exclusion criteria were: an acute relapse within the previous 3 months and another central neurological disease. In total, 606 patients with MS with 5117 visits were made between January 2017 and September 2021, out of them 258 patients with MS and 572 visits were enrolled in this study. All patients fulfilled the criteria of MS, according to the 2017 McDonald criteria (21).

### Statistical Analysis

We investigated three clinically plausible hypotheses regarding the time-dependency of fatigue over 1 year: (1) no time dependency; (2) a linear trend over time, or (3) a non-linear trend over time. Accordingly, we constructed different regression models that reflected the three hypotheses, as follows:

(1) No Time Dependency:

- NULL model: Fatigue score – BDI + EDSS + SDMT + age + sex

(2) Linear Time Dependency:

- NULL model + months

(3) Non-Linear Time Dependency:

- NULL model + months + months^2^
- NULL model + months + months^3^
- NULL model + months^2^ + months^3^
- NULL model + months^2^
- NULL model + months^3^

To determine which model provided the best description of our data, we applied an information theory-based model-selection approach, based on Akaike's information criterion (AIC) (22). The model with the smallest AIC had the highest support from the data.

We calculated the following parameters:


(1) $$\begin{array}{l} \text{AIC difference}:\;{\Delta \text{AIC}}_{i}=\;{\text{AIC}}_{i}-{\text{AIC}}_{\text{min}} \end{array}$$

(2) $$\begin{array}{l} \text{Akaike weight:}w_{i}=\frac{\text{exp}\left(-0.5\cdot {\Delta \text{AIC}}_{i}\right)}{\sum_{r=1}^{R}\text{exp}\left(-0.5\cdot {\Delta \text{AIC}}_{r}\right)}\;\text{and} \end{array}$$

(3) $$\begin{array}{l} \text{Evidence ratio: }\text{ER}=\frac{\text{exp}\left(-0.5\cdot {\Delta \text{AIC}}_{\text{best}}\right)}{\text{exp}\left(-0.5\cdot {\Delta \text{AIC}}_{i}\right)} \end{array}$$

The Akaike weight can be interpreted as the conditional probability that the current model (*i*) is the best model of the set. The evidence ratio provides a measure of how much more likely the best model (*best*) is, compared to the current model (*i*). We used the linear mixed-effects model approach (-xtmixed-) provided in Stata statistical software® (Version 17.1, Stata Corp, College Station, TX, USA) to model the time course over 12 months of a year. Patient-ID and year were considered random factors, because we had repeated visits by patients and several years of follow-up. All models were adjusted for the baseline covariables, age, sex, and possible interacting variables BDI, EDSS and SDMT. *P*-values < 0.05 were considered statistically significant.

In a second step, Spearman's rank correlation was performed to asses the relationship between the mean FSMC per month and the average monthly outdoor temperature. Therefore, the mean outdoor temperature in Mecklenburg-Vorpommern during 01/2017 and 09/2021was also added according to the information from the Deutsche Wetterdienst (DWD).

## Results

### Patient Characteristics

We enrolled 258 patients with MS (176 females, 82 males) and analyzed 572 visits in this study (see Table 1). The mean age at the baseline visit was 42.09 years (SD: 12.24), the mean BDI was 9.12 (SD: 8.48), the mean SDMT was 47.38 (SD: 13.59), and the median EDSS was 2.0 (range: 0–8).

**Table 1:** Patients' characteristics.

		**n**	**Mean**	**SD**
Patients		258		
	Sex (f/m)	176/82		
	Age at baseline (y)		42.09	12.24
	Disease duration at baseline (y)		9.41	7.58
	Disease course at baseline (RRMS/SPMS/PPMS)	198/41/19		
	DMT at baseline Glatiramer acetate Interferon beta Fingolimod Dimethyl fumarate Teriflunomide Ozanimod Siponimod Cladribine Ocrelizumab Natalizumab Alemtuzumab None	31 28 37 36 23 2 3 5 11 25 11 46		
Visits		572	0.130	1.806
	EDSS (median/range)		2	0–8
	BDI		9.12	8.48
	SDMT		47.38	13.59

### Fatigue Scores

The mean number of FSMC scores per patient was 2.2 (range: 1 to 5 scores per patient). The mean number of FSMC scores per month was 47.7 (range: 31 in April to 65 in January and June). The minimum and maximum fatigue scores were documented, respectively, during visits in January (mean FSMC: 49.84) and August (mean FSMC: 53.88, Table 2).

**Table 2:** Mean fatigue scores (FSMC), outdoor temperature (°C) and the number of datapoints per month for patients with MS.

**Month**	**N**	**FSMC mean**	**95% CI**	**Mean temperature**
January	65	49.84	46.10–53.57	2.0
February	32	50.97	47.46–54.47	2.2
March	39	51.91	48.47–55.35	4.6
April	31	52.67	49.20–56.14	8.4
May	45	53.25	49.73–56.77	12.6
June	65	53.64	50.10–57.18	18.0
July	48	53.85	50.33–57.37	18.3
August	45	53.88	50.42–57.33	18.6
September	58	53.72	50.34–57.11	14.6
October	39	53.38	50.01–56.75	11.1
November	64	52.86	49.36–56.36	6.0
December	41	52.15	48.30–56.01	3.9

### Regression Models

**Figure 1:**
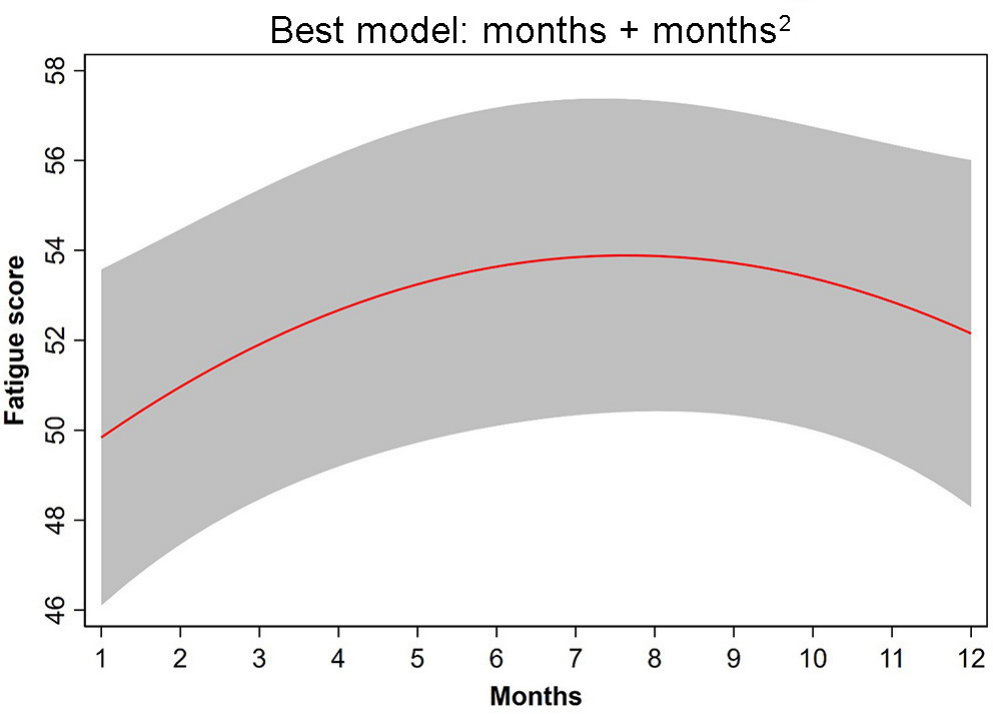
The non-linear time-dependency regression NULL model + months + months^2^ provided the best fit to the fatigue data.

Among the hypothetical regression models, the non-linear time dependency model: NULL + months + months^2^ fit the data best (Table 3). In this model, the parameters, month (β = 1.402; CI = 0.229, 2.505) and month^2^ (β = −0.092; CI = −0.178, −0.005), had significant effects (*p* = 0.013, *p* = 0.038, respectively). This model revealed that fatigue increased significantly from June to September (Figure 1).

**Table 3:** Regression models constructed to investigate the time-dependency of fatigue in MS.

**Model name**	**df**	**AIC_**i**_**	**ΔAIC**	**Aweight**	**ER**
***NULL** **+** **months** **+** **months**^**(2)**^*	***11***	***4523.639***	***0.000***	***0.234***	***——***
NULL + months + months^(3)^	11	4523.960	0.321	0.199	1.174
NULL + months	10	4524.112	0.474	0.185	1.267
NULL + months^(2)^ + months^(3)^	11	4524.821	1.182	0.130	1.806
NULL + months^(2)^	10	4525.290	1.651	0.103	2.283
NULL	9	4525.820	2.181	0.079	2.976
NULL + months^(3)^	10	4526.016	2.377	0.071	3.283

### Correlation Between Fatigue Score and Outoor Temperature

Spearman correlation revealed a significant relationship between the mean FSMC per month and the average monthly outdoor temperature (ρ = 0.972; *p* < 0.001).

## Discussion

This was the first study to demonstrate, in a systematic way, that fatigue had a temporal course in MS. We found that fatigue increased during summer and decreased during winter. Moreover, this fluctuation was not explained by age, sex, disease severity, depression, or cognition.

Previous studies have shown that MS-related fatigue increased with ambient heat, based on different patient questionnaires (4, 16). To our knowledge, only one previous study conducted a longitudinal investigation of seasonal fluctuations in fatigue in relation to outdoor temperature among patients with MS (17). Those authors used a 7-point scale to rate fatigue in a cohort of 45 Greek patients with MS. However, they did not find a significant difference in symptom severity between February, May, August, and November. In contrast, the present study used the validated FSMC to assess fatigue in a large cohort of real-world patients with MS in Germany. With this approach, we identified seasonal fluctuations. The peak fatigue was observed in August (mean FSMC-score: 53.88) and the minimum fatigue was observed in January (mean FSMC-score: 49.84). The discrepancy between our study and the study conducted by Bakalidou et al. (17) might have been due to methodological differences, as our sample was larger (*n* = 258 vs. *n* = 45), we used an international, validated scale for assessing fatigue (FSMC vs. a 7-point Likert scale), and we performed assessments more often (12 vs. 4 time-points per year), compared to the study by Bakalidou et al. Furthermore, the increasing FSMC-score during the summer occurred simultaneously with the rising outdoor temperature, suggesting its causal relationship. In the study conducted in Greece, the mean difference between February and August temperatures was 18.5°C. With this difference, they could not find any seasonal fluctuation in fatigue. In contrast, in the area of Mecklenburg-Vorpommern, where the present study was conducted, the mean seasonal difference in temperature was 15.6 °C (23), and 16.6 °C during the observed period of time, which was less than the seasonal fluctuation observed in the study by Bakalidou et al. (15). Therefore, we detected a seasonal difference in fatigue, despite less fluctuation in outdoor temperature. The temporal association should be validated in different cohorts, especially in areas with different temperature levels.

Fatigue has both objective and subjective aspects (11). Objective variables, like the MS disease, cannot be changed. However, subjective variables, like mood, cognition, motivation, or activity levels, might be influenced by environmental conditions, like outdoor temperature. We demonstrated that fatigue showed seasonal fluctuations, even after we controlled for the main clinical variables of individual patients, including age, sex, disability, depression, and cognition. That result suggested that outdoor temperature may have an impact on fatigue in patients with MS. Nevertheless, it remains unclear whether this impact is due to a direct effect of the outdoor temperature on body temperature, where an influence on fatigue could be shown (24), or the direct sun exposure (25), or whether it is an indirect effect of homeostatic factors that are also related to fatigue (6, 10, 11). Some authors also define a metacognitive concept of fatigue to explain the subjective experience of fatigue (6), which both might also be influenced by temporal factors like temperature. Alternatively, the association between subjective fatigue and outdoor temperature might also be due other moderating or interacting variables, which have to be considered in future investigations. We here could not determine the underlying causes of fatigue, because we only evaluated seasonal changes. However, we did control for interacting factors like neuropsychological symptoms, cognition, and depression (12). Therefore, we could assume that the variation in fatigue was not caused by simple variations in these variables.

This study had several limitations. First, the design of the study was retrospective. However, the data represent real-world data from the outpatient clinics, where all variables were collected during routine consultations. However, all the applied measures were well-known, validated screening tools with high sensitivity, for example, the information processing speed (26). Second, we may not have included confounders like sleep quality. Thus, future prospective studies should include more detailed information. Third, in this cohort study, we only measured 1 to 5 time-points per patient. Future studies should be designed longitudinally, with more time-points, to confirm the associations described in our study. Finally, we did not include a control group. Therefore, we could not exclude the possibility that the seasonal fluctuation in fatigue might have been detectable, independent of the MS disease. Future prospective studies should include a control group to provide a comparison of the fluctuations in fatigue between healthy participants, MS patients and patients with various disease conditions, which also increases the knowledge about the MS specific Uhthoff's phenomenon (13).

In conclusion, we demonstrated that fatigue was modulated temporally throughout the year. This seasonal fluctuation, with an increase in fatigue during the summer, should be taken into account in the assessment for fatigue in patients with multiple sclerosis. In addition, in therapeutic research, this seasonal fluctuation and its association to outdoor temperature should be considered a potential confounding factor when evaluating therapeutic effects in patients with MS.

## Data Availability Statement

The raw data supporting the conclusions of this article will be made available by the authors, without undue reservation.

## Ethics Statement

The studies involving human participants were reviewed and approved by Ethics Committee Universitätsmedizin Greifswald. The patients/participants provided their written informed consent to participate in this study.

## Author Contributions

MG, SG, and IP contributed to the conception and design of the study and analyzed the data. MG and IP wrote the manuscript. SG, MS, and SS provided feedback and edited the manuscript. All authors contributed to the article and approved the submitted version.

## Funding

This research was funded by a research grant from Novartis. The funders played no role in the design of the study, in the collection, analyses, or interpretation of data, in the writing of the manuscript, or in the decision to publish the results.

## Conflict of Interest

MG received honoraria and travel reimbursements for attending meetings, from Biogen, Celgene, Merck Serono, Novartis, Roche, Sanofi Genzyme, and TEVA. His research is funded by the German Ministry for Education and Research BMBF, Merck Serono, and Novartis. None of these relationships resulted in a conflict of interest. IP has received honoraria for speaking at scientific meetings, serving at scientific advisory boards, and performing consulting activities, from Adamas Pharma, Almirall, Bayer Pharma, Biogen, BMS, Celgene, Desitin, Sanofi-Genzyme, Janssen, Merck, Novartis, Roche, and Teva. She received research support from the German MS Society, Celgene, Novartis, Roche, and Teva. None of these relationships resulted in a conflict of interest. MS received honoraria for attending meetings, from Biogen and Merck Serono. None of these relationships resulted in a conflict of interest. The remaining authors declare that the research was conducted in the absence of any commercial or financial relationships that could be construed as a potential conflict of interest.

## Publisher's Note

All claims expressed in this article are solely those of the authors and do not necessarily represent those of their affiliated organizations, or those of the publisher, the editors and the reviewers. Any product that may be evaluated in this article, or claim that may be made by its manufacturer, is not guaranteed or endorsed by the publisher.
